# Presence of a Chaotic Region at the Sleep-Wake Transition in a Simplified Thalamocortical Circuit Model

**DOI:** 10.3389/fncom.2016.00091

**Published:** 2016-09-01

**Authors:** Kush Paul, Lawrence J. Cauller, Daniel A. Llano

**Affiliations:** ^1^Department of Molecular and Integrative Physiology, University of Illinois at Urbana-ChampaignUrbana, IL, USA; ^2^Beckman Institute for Advanced Science and Technology, University of Illinois at Urbana-ChampaignUrbana, IL, USA; ^3^School of Behavioral and Brain Sciences, University of Texas at DallasRichardson, TX, USA

**Keywords:** thalamocortical, computational model, nonlinear dynamics, GENESIS, chaos

## Abstract

Sleep and wakefulness are characterized by distinct states of thalamocortical network oscillations. The complex interplay of ionic conductances within the thalamo-reticular-cortical network give rise to these multiple modes of activity and a rapid transition exists between these modes. To better understand this transition, we constructed a simplified computational model based on physiological recordings and physiologically realistic parameters of a three-neuron network containing a thalamocortical cell, a thalamic reticular neuron, and a corticothalamic cell. The network can assume multiple states of oscillatory activity, resembling sleep, wakefulness, and the transition between these two. We found that during the transition period, but not during other states, thalamic and cortical neurons displayed chaotic dynamics, based on the presence of strange attractors, estimation of positive Lyapunov exponents and the presence of a fractal dimension in the spike trains. These dynamics were quantitatively dependent on certain features of the network, such as the presence of corticothalamic feedback and the strength of inhibition between the thalamic reticular nucleus and thalamocortical neurons. These data suggest that chaotic dynamics facilitate a rapid transition between sleep and wakefulness and produce a series of experimentally testable predictions to further investigate the events occurring during the sleep-wake transition period.

## Introduction

The specific anatomy of thalamocortical circuitry along with the interactions of ionic conductances in thalamic and cortical cells give rise to multiple modes of activity that characterize behavioral states such as the sleep-wake cycle and generalized epilepsy. During drowsiness and sleep, large groups of neurons fire synchronously giving rise to oscillatory activity such as spindle (6–14 Hz), delta (1–4 Hz), and slow (<1 Hz) oscillations, all of which may be observed in the constituent cells of the thalamus and cortex; thalamocortical (TC) cells, neurons in the thalamic reticular nucleus (RE) and deep-layer cortical neurons (CX; Steriade, 2005; Huguenard and McCormick, 2007). TC neurons project primarily to layer 4 of cortex, and also send projections to other layers, including infragranular layers (Beierlein and Connors, 2002; Meyer et al., 2010). Corticothalamic projections, which arise from layers 5 to 6, terminate on TC neurons as well as on neurons of the RE (Liu and Jones, 1999; Zikopoulos and Barbas, 2006; Llano and Sherman, 2008). TRN neurons, which receive excitatory input from TC and CX neurons, send GABAergic projections to TC neurons (Pinault, 2004). Interplay of intrinsic ionic properties of single neurons from these structures, modulated and orchestrated by the synaptic interactions of large groups of neurons, likely play a large role in generating coherent oscillatory activity in the brain.

One cellular mechanism which has been postulated to play an important role for the generation of large-scale oscillatory activity is post-inhibitory rebound (PIR) present in TC and RE neurons, as well as a subset of CX neurons (Stafstrom et al., 1984; Steriade et al., 1993b). This mechanism is associated with low-threshold Ca^2+^ (I_T_) as well as hyperpolarization-activated cation currents (I_H_) through hyperpolarization-activated cyclic nucleotide-gated (HCN) channels (Lüthi and McCormick, 1998; Sherman, 2001; Timofeev et al., 2002; Kramer et al., 2008). For the calcium component, after TC neurons have been hyperpolarized for a protracted period of time (~50–100 ms), I_T_ becomes de-inactivated, releasing the low-threshold calcium conductance for activation (Jahnsen and Llinas, 1984). Elimination of I_T_ greatly disrupts spindle and absence seizure activity (Kim et al., 2001; Astori et al., 2011), suggesting that this current is critical for producing synchronized oscillations in the thalamus. For I_H_, after being activated by hyperpolarization, the return to baseline voltage is associated with delayed closure of HCN channels, leading to rebound depolarization (Pape, 1996; Slater et al., 2013). The combined effect of these two currents is typically a low-threshold slow rebound depolarization with a superimposed burst of traditional sodium action potentials (Lüthi and McCormick, 1998). In the context of a network containing TC, RE, and CX cells, this post-inhibitory rebound can be triggered after TRN-based GABAergic inhibition in TC cells, producing a TC burst, which would produce excitation in a RE cell. Such a cycle may be initiated by cortical activation of the thalamic reticular nucleus, and can generate oscillations associated with sleep spindles (Steriade, 2005) as well as paroxysmal activity (Meeren et al., 2002; Polack et al., 2007).

What is still unclear is the nature of the state transitions between sleep and wakefulness that are characterized by marked difference in thalamocortical oscillatory behavior. Slow-wave sleep is characterized rhythmic, delta-range bursting in TC cells (Amzica and Steriade, 1998), while the waking state is dominated by irregular isolated action potentials and occasional bursts (Ramcharan et al., 2000; Bezdudnaya et al., 2006). Previous work in relatively simple models has suggested that TC cells near the onset of rhythmic bursting show chaotic behavior (Wang, 1994; Paul et al., 1998); a type of dynamical interaction which may facilitate the rapid transition between discrete states in neural systems (van Vreeswijk and Sompolinsky, 1996). Therefore, the objective of the present study was to characterize state transitions using tools of nonlinear dynamics in a reduced but biologically realistic model of the thalamo-reticulo-cortical circuit that maintains the essential intrinsic conductances in these nuclei and their synaptic connectivity. We assume here that transitions from slow oscillatory behavior seen in model TC neurons to rapid, irregular firing corresponds to a transition between sleep and wakefulness.

The traditional linear spike train analyses treat variability in a biological system as noise and average out this factor. This approach does not take into account that this variability may be an inherent property of an interconnected dynamical system such as the thalamocortical system. Chaos refers to deterministic or non-random behavior which exhibits very rapid growth of errors leading to different system states and thus renders impossible any long-term prediction. Others have argued that the irregularity of spike patterns in large networks is an emergent property of the network that enables it to respond very rapidly to external input, much faster than the time constant of individual neurons, and therefore benefits neural processing (van Vreeswijk and Sompolinsky, 1996; Bertschinger and Natschläger, 2004; Canavier and Shepard, 2009; Sussillo and Abbott, 2009). Previous studies have shown that artificial neuronal networks and real networks of neurons can demonstrate the presence of chaotic regimes (Canavier et al., 1990; Wang, 1994; van Vreeswijk and Sompolinsky, 1996; Siegel and Read, 2001; Bertschinger and Natschläger, 2004; Battaglia et al., 2007; Sussillo and Abbott, 2009; Rajan et al., 2010; Jia et al., 2012). Other studies have demonstrated fractalness in spike trains (Teich, 1989; Teich et al., 1997; Darbin et al., 2006; Gebber et al., 2006). These studies suggest that chaotic dynamics exist in a large number of simulated and real neural systems.

**Figure 1:**
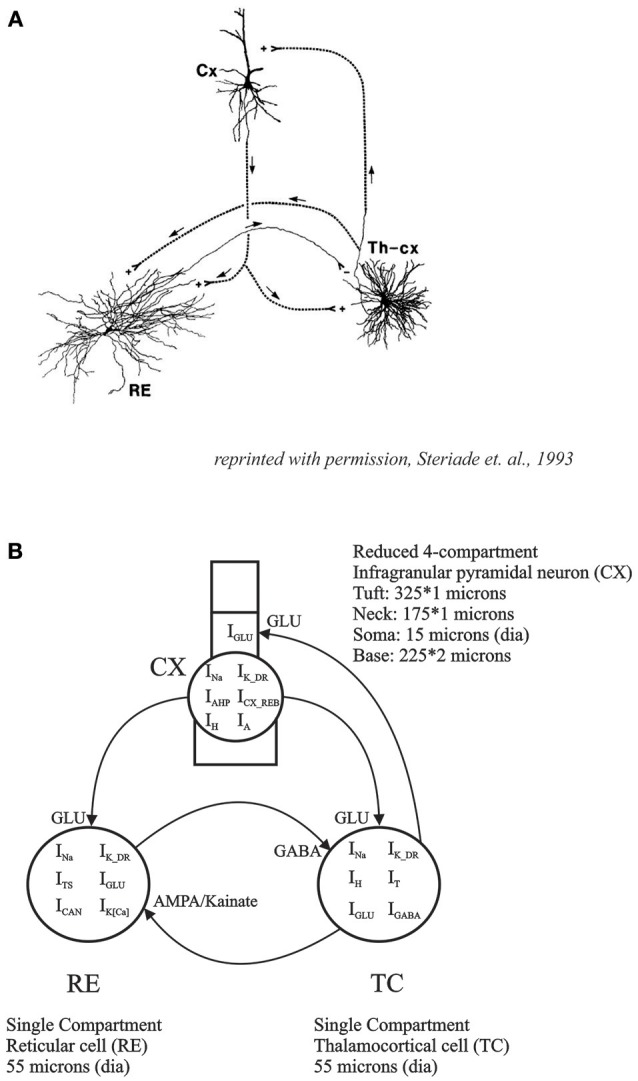
**(A)** Basic thalamo-cortical circuit consists of reciprocal excitatory connections between TC and CX neurons with collaterals to RE. RE neurons have inhibitory projections to TC (reprinted with permission, Steriade et al., 1993a). **(B)** Thalamo-Reticulo-Cortical network model developed for simulations in the current study. This computational model with anatomically realistic connections is an implementation of the proposed circuitry for the neural basis of thalamocortical oscillations.

In the present study, we have constructed a reduced 3 neuron thalamo-reticulo-cortical model consisting of one CX corticothalamic neuron, one TC neuron and one RE neuron with intrinsic conductances, based in part on our intracellular recordings, and biologically realistic synaptic connectivity between them. The model is a direct implementation of the circuitry that has been hypothesized to be essential for thalamocortical oscillatory behavior (Steriade et al., 1993a,b; Steriade, 2005; Contreras, 2014; see Figure 1). The implementation of post-inhibitory rebound and associated low-threshold Ca^2+^ conductance was based on *in vitro* data from cortical slices and then fit to the model to obtain realistic rebound excitation in an infragranular CX neuron. In the interconnected model, we demonstrated periodic oscillatory and irregular aperiodic spike burst patterns depending on the strength of synaptic connectivity.

Finally, we demonstrate that within a range of synaptic conductance values representing putative physiological transition states, chaotic behavior in the network model is obtained.

## Methods

### Electrophysiology

All procedures were performed under the guidelines of the National Institutes of Health and approved by the Institutional Animal Care and Use Committee. Adult male rats (100–250) g were anesthetized with sodium pentobarbital and decapitated and the brain quickly removed and put in ice-cold artificial cerebrospinal fluid (ACSF) bubbled with 95% O_2_—5% CO_2_. The composition of ACSF was in mM NaCl (126), KCl (3), MgSO_4_ (1), NaH_2_PO_4_ (1.25), NaHCO_2_ (26), Glucose (10), and CaCl_2_ (2). Coronal slices (400 μm) were prepared through the primary somatosensory area and placed in an interface type slice chamber with a continuous flow of ACSF maintained at a temperature of (37 ± 1)°C. Somatosensory cortex was used since oscillatory phenomena that accompany states of sleep and drowsiness involve large regions of the brain and are found throughout the brain and because spindle oscillations that accompany drowsiness and sleep are found in the somatosensory cortex (Contreras and Steriade, 1995; Khazipov et al., 2004; Rosanova and Timofeev, 2005; Halassa et al., 2011). Blind intracellular recordings employed sharp micropipettes (Brown Flaming Puller; 100–160 MΩ) filled with 1M K-acetate (pH 7.4). Membrane potentials were recorded with an Axoprobe-1A amplifier. The output was displayed on a digital storage oscilloscope and stored on a videocassette recorder via a Neuro-corder (Model DR-890, Neurodata Instruments) for offline analysis. Analysis was done using custom written software (Dataman PC, Cauller). Neurons in infragranular layers V/VI were classified as either non-adapting or adapting based upon their response to sustained somatic current injection.

### Computational model

A reduced cortical infragranular pyramidal cell model was developed in the GENESIS simulation environment (cortical infragranular cell, CX, in Figure 1). It consisted of four compartments: tuft, neck, soma and base. The tuft (325 μm length and 1 μm diameter) and neck (175 × 1 μm) together represent the apical dendrite. The neck was attached to the apical side of the soma (15 × 15 μm) whereas the base (225 × 2 μm) represented the basal dendrites. The membrane resistivity (R_M_), axial resistivity (R_A_) and membrane capacitivity (C_M_) were set at 0.5 Ω/m^2^, 1.0 Ω/m, and 0.017 F/m^2^, respectively. The model consisted of the following currents: fast sodium (I_Na_), potassium delayed rectifier (I_K_DR_), cortical low-threshold/rebound (I_CX_REB_), hyperpolarization-activated cation (I_H_), potassium after-hyperpolarization (I_AHP_), calcium dependent potassium (I_K[Ca]_), and the potassium A-current (I_A_). The channel description for the I_CX_REB_ current was implemented based on data directly obtained from the activation and inactivation curves obtained from *in vitro* experiments described above. All other channel descriptions were taken from existing GENESIS user libraries. The maximal conductance values used were (in S/m^2^): *g*_K_DR_ = 200, *g*_CX_REB_ = 7, *g*_H_ = 12.55, *g*_AHP_ = 2.1, *g*_K[Ca]_ = 0.28, and *g*_A_ = 12. To determine rebound parameters in isolation, the fast Na^+^ maximal conductance was set to zero (*g*_Na_ = 0) to simulate the effect of the intracellular Na^+^ blocker QX-314, as was done in physiological experiments.

The differential equation governing the membrane voltage of the model was given by

$$\begin{array}{lll} C\frac{{dV}_{CX}}{dt} & = & -\left(I_{Na}+I_{K_{DR}}+I_{CX_{REB}}+I_{H}+I_{AHP}+I_{A}+I_{K\left[{Ca}^{2+}\right]}\right)\\ I_{j} & = & {\bar{g}}_{j}m^{M}h^{N}\left(V_{CX}\;-\;E_{j}\right) \end{array}$$

Here, *C* is the capacitance of the membrane, *V*_*CX*_ is the membrane voltage and I_*j*_ is the current passing through each specific channel. *g*_j_ is the maximal conductance value for each channel (Bower and Beeman, 1998, Chapters 4 and 6).

*m* and *h* represent the instantaneous activation and inactivation variables. They are raised to the power *M* and *N*, respectively. *E*_*j*_ is the reversal potential of each channel *j*. The steady state activation and inactivation variables *m*_∞_ and *h*_∞_ were either obtained from experimental data or from equations fitted to experimental data as described above. The instantaneous values of these gates change with respect to time as

$$\begin{array}{l} m_{\infty },h_{\infty }=\frac{\alpha }{\alpha +\beta }\\ \;\frac{dm}{dt}=\frac{\left(m_{\infty }-m\right)}{\tau_{m}},\frac{dh}{dt}=\frac{\left(h_{\infty }-h\right)}{\tau_{h}} \end{array}$$

Where, τ_*m*_ and τ_*h*_ are the activation and inactivation time constants, α and β are the forward and backward rate constants, respectively (Bower and Beeman, 1998).

From first-order kinetics, α and β are of the form

$$\begin{array}{l} \alpha ,\beta =\frac{A+Bx}{C+e^{\frac{V_{CX}+D}{F}}} \end{array}$$

Where, *A–F* are constants and *V*_*CX*_ is the membrane potential of the cortical neuron.

For Ca^2+^ dependent processes, α and β are multiplied by a factor representing the intracellular Ca^2+^ concentration. Change in the intracellular *Ca*^2+^ concentration is described by

$$\begin{array}{l} \frac{d\left[{Ca}^{2+}\right]}{dt}=B.I_{{Ca}^{2+}}-\frac{\left[{Ca}^{2+}\right]}{\tau_{{Ca}^{2}+}} \end{array}$$

Here, τ_*Ca*_ is a factor representing the rate of decay of [*Ca*^2+^]. *B* is a factor representing the flux of ions in a thin shell near the membrane surface produced by *I*_*Ca*_ (Bower and Beeman, 1998, Chapter 19).

The synaptic conductance change is modeled by the “alpha function” (Rall, 1967).

$$\begin{array}{l} g_{syn}\left(t\right)=g_{max}\frac{t}{t_{p}}e^{\left(1-t/t_{p}\right)} \end{array}$$

Where, *g*_*syn*_ is the instantanteous synaptic conductance value of either of g_GLU_ or g_GABA_. At *t* = *t*_*p*_, the function rises to a maximal conductance value of *g*_*max*_ for each of the synaptic conductances and then decreases slowly to zero. The synaptic current is then given as

$$\begin{array}{l} I_{syn}\left(t\right)=g_{syn}\left(t\right)\left(V_{m}-E_{syn}\right) \end{array}$$

Where *V*_*m*_ is the membrane potential and *E*_*syn*_ is the synaptic reversal potential of the synaptic conductance (i.e., *E*_*GLU*_ *or E*_*GABA*_).

For the network model, the simplified 4 compartment model of an infragranular CX cell was synaptically connected to the thalamo-reticular (TC-RE) model (Paul et al., 1998). The TC cell model included I_T_, I_H_, I_Na_, I_K_, and I_GABA_ and the RE model consisted of I_TS_, I_K[Ca2+]_, I_CAN_, I_Na_, I_K_, and I_AMPA_ to implement the three neuron network TC-RE-CX model (Figure 1). This network model is an implementation of the Steriade (Steriade et al., 1993b,c; Steriade, 2005) hypothesis for the neural basis of oscillations in the thalamus and the cortex. The TC-RE network has reciprocal excitatory and inhibitory connections. The TC cell has excitatory AMPA/Kainate projections to the RE cell which, in turn, has GABAergic inhibitory projections to the TC. The CX cell is connected to this network with excitatory glutamate projections to both TC and to RE. The TC cell has reciprocal excitatory projections to the neck compartment of CX. This approximates the thalamocortical excitatory projections at the proximal apical dendrites (layer IV synapses). The maximal conductance values for the CX cell was slightly adjusted to obtain oscillatory behavior in the CX cell in concert with those in the TC and RE cells. These values are given in Table 1. The network model was constructed such that RE acted as the initiator or pacemaker cell, based on findings from lesion experiments in the cat (Steriade et al., 1987).

**Table 1:** **Maximal intrinsic conductance values for each compartment of the network model**.

**Cell Compartment**	**Maximal Intrinsic Ionic Conductances (S/m^2^)**
CX_soma	gNa = 2000
	gK_DR = 200
	gCX_REB = 10
	gH = 12.55
	gAHP = 2.1
	gK[Ca^2+^] = 0.28
	gA = 12
CX_neck	gCX_REB = 4
TC	gNA = 1737
	gK_DR = 206.9
	gT = 40
	gH = 10.55
	gLEAK = 3
RE	gNa = 1737
	gK_DR = 256.9
	gTs = 250.61
	gCAN = 1.5
	gK[Ca^2+^] = 3.5

### Nonlinear dynamical analysis

**Figure 2:**
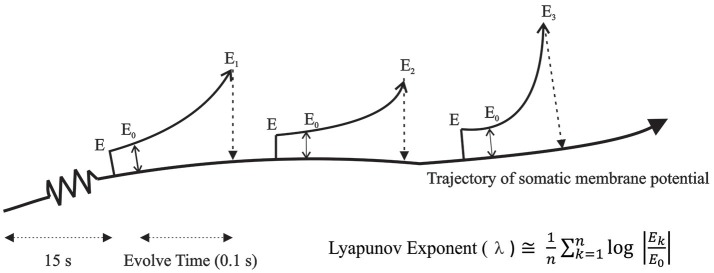
**Algorithm for computing Lyapunov Exponents**. Two identical networks of the TC-RE-CX reciprocally connected model were created and an error was introduced in the membrane potential of the copy system and allowed to amplify for a period of time (0.1 s) after which the difference between the original system and the copy system was measured and all parameters of the copy system was reset to the values of the original system. The average rate of divergence or convergence was then computed using the formula shown in the figure.

To identify chaotic regimes within the dynamic system four methods of analyses were used. These are elaborated below:

*Phase space analysis:* Two time-dependent variables were plotted against each other over time. Such a plot provides a graphical picture of the dynamical system being investigated and differentiates between a fixed point attractor, a limit cycle and strange attractors. Presence of a strange attractor suggests the presence of a chaotic regime within the dynamical system.
*Bifurcation plots:* A bifurcation plot illustrates the transition from periodic to aperiodic or chaotic states thus plotting the transition from order to chaos in a dynamical system. These are usually plotted with a system parameter on the x-axis and a representation of an attractor on the y-axis. At a bifurcation point, the attractor in the plot splits into two if the attractor changes from a period of one to a period of two.
*Fractal dimension:* Two measures were used to compute the fractal dimension of neuronal spike train pattern. These are the Fano Factor and the Allen Factor. Fano Factor refers to the ratio of the variance in the number of spikes within a time window T to the mean number of spikes within that same time window. This is given as
  $$\begin{array}{l} F\left(T\right)=\frac{Var\left[N\left(T\right)\right]}{\langle N\left(T\right)\rangle } \end{array}$$
  Experimentally, the Fano Factor is calculated by computing the above metric repeatedly for different time windows (*T*) over the range of interest. For a true Poission process, *F(T)* is equal to 1. *F(T)* > 1 imply that the variance in the number of events grows faster than the mean, suggesting the presence of long-term correlations in the data and fractal properties (Teich, 1989; Lowen and Teich, 1996; Koch, 1999). Due to the nature of computation of the Fano Factor, it cannot increase faster than ~T^1^ (Lowen and Teich, 1996). Therefore, a second measure, the Allan Factor, which has been used to detect self-similarity with fractal exponents greater than unity (Allan, 1966; Barnes and Allan, 1966), was used here. The Allan Factor is derived from the Allan variance which is given as the average variation in the difference of adjacent counts. The Allan Factor is then the ratio of the Allan variance of the even count to twice the mean. It is related to the Fano Factor by A(T) = 2F(T) – F(2T) where, *F(T)* is the Fano Factor for counting time T (Scharf et al., 1995). The Allan Factor can rise as fast as ~T^3^ and therefore, can be used to estimate fractal exponents in the range 0–3 (Lowen and Teich, 1996).
*Lyapunov exponents:* The method for computing Lyapunov Exponents involved a direct approach which was possible within a simulation environment. This algorithm is graphically shown in Figure 2. Two identical networks of the TC-RE-CX reciprocally connected model were created and were run for a period of 15 s simulation time to eliminate transients. An iteration time step (dt) of 10 μs was used. After 15 s of simulation run time, a perturbation of 0.005 mV introduced into the copy of the original system. The two parallel simulations were now run for 5 time steps to allow the initial perturbation introduced into the membrane voltage to filter through to all other parameters. The error E0 was measured at this time and the simulations were run for an evolve time of 0.1 s during which the introduced “error” was allowed to amplify (Ek). The parameter of interest was the somatic membrane potential which was recorded from both the original system and the perturbed copy and the log of the absolute difference (“error”) was computed. All parameters of the perturbed system were reset to the values of the original system and this procedure was repeated for 1000 iterations. The average rate of divergence or convergence (i.e., Lyapunov exponent) was then computed using the formula:
  $$\begin{array}{l} \lambda ≅\frac{1}{n}\overset{n}{\underset{k=1}{\sum }}\log|\frac{E_{k}}{E_{0}}| \end{array}$$
  This procedure of perturb, run, collect data, reset and perturb again prevented the errors from becoming unbounded, therefore enabling the algorithm to detect any local “stretches” while avoiding the global “fold” (Paul et al., 1998).

For all methods (except Lyapunov Exponents), the analysis was done over 20 s of data after removal of the first 5 s in order to avoid the initial transients. The characterization of network activity as periodic or aperiodic was based on the spike pattern of the CX cell. The two parameters that were systematically varied while computing the Lyapunov exponent were the strength of TC → CX synaptic excitation and the strength of RE → TC synaptic inhibition.

Two representative data sets are used for detailed analysis for each of the methods to distinguish between periodic state (as observed during sleep) and chaotic state (transition state). Additionally, the bifurcation plot and Lyapunov exponents were computed for a range of data points that demonstrate a transition from one mode to the other. Physiologically, transition from sleep to wakefulness was simulated by decreasing a potassium leak conductance (*g*_LEAK_) to the TC and RE cells from its initial setting. This approach has been used previously in thalamic (Bazhenov et al., 2002; Willis et al., 2015) and cortical (Hill and Tononi, 2005) models, and simulates excitatory brainstem input (e.g., cholinergic) that accompanies the transition between these behavioral states and modulates the resting membrane potential of these cells. Lyapunov Exponents were computed for a range of *g*_LEAK_ values.

## Results

### *In vitro* determination of cortical neuron post-inhibitory rebound parameters

**Figure 3:**
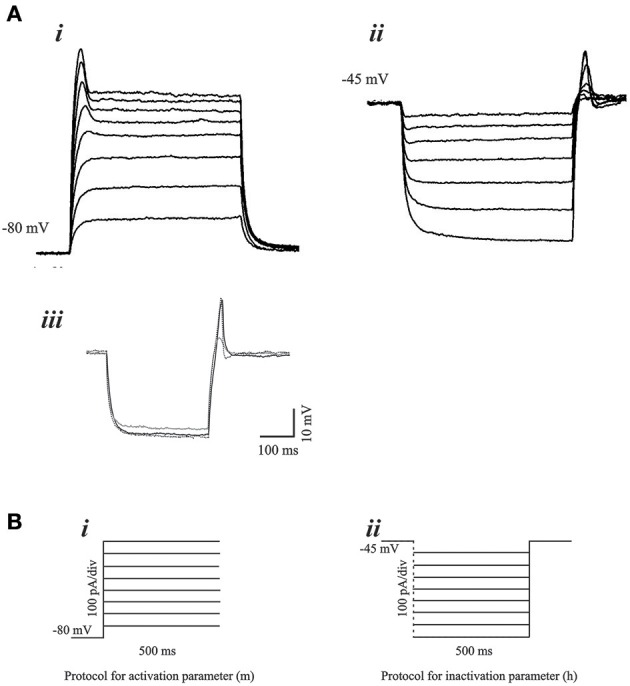
**Voltage responses to current stimulus protocols**. **(A)** Typical voltage responses to current stimulus for ***(i)*** activation and ***(ii)*** inactivation of the low-threshold conductance. Blockade of PIR response by Co^2+^ ***(iii)***, demonstrates the Ca^2+^ dependence of the PIR response. **(B)** ***(i)*** Experimental protocol for determination of the activation parameter consisted of 500 ms step pulses of increasing current amplitude. The holding current was adjusted such that the membrane potential was at −80 mV at the start of the step pulse. ***(ii)*** Protocol for determination of the inactivation parameter consisted of hyperpolarizing current steps of increasing amplitude and of 500 ms duration. The holding current at the start of the step pulses is adjusted such that the membrane potential is at −45 mV.

We examined PIR properties mediated by low threshold Ca^2+^ conductance (I_T_) in real infragranular cortical neurons in brain slices. These neurons are hypothesized to contribute to the dynamic behavior of cortico-thalamic networks and PIR plays a critical role in network oscillations. The rebound depolarization properties obtained from *in vitro* experiments provide the activation and inactivation variables for the implementation of I_T_ conductance in infragranular cortical neurons. PIR is mediated by a low threshold calcium conductance or low threshold calcium spike (LTS). The mean resting membrane potential of the rebound cells was −65.56±4.27 mV. In the presence of intracellular blocker QX-314 (50 mM), the mean LTS amplitude 13.57 ± 4.05 mV, and the LTS width was 67.6 ± 21.55 ms. Figures 3A*i,ii* demonstrates typical voltage responses to current stimuli in a neuron for each of the protocols used to obtain low-threshold conductance properties, namely, the activation parameter (*m*), and the inactivation parameter (*h*). The Ca^2+^ dependence of the post-inhibitory rebound is shown by block and recovery with Co^2+^ (Figure 3A*iii*). The current stimulus protocols used for obtaining the voltage responses for activation and inactivation parameters are shown in Figures 3B*i*,B*ii*, respectively.

**Figure 4:**
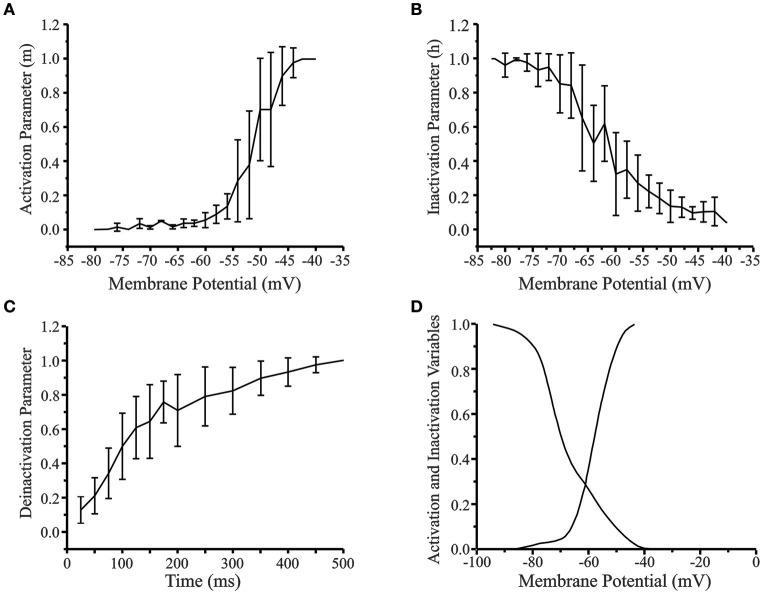
**(A)** Activation curve of the low-threshold conductance in cortical layer V neurons (*n* = 30). **(B)** Inactivation curve. **(C)** Time course of deinactivation. Standard error is indicated for each data point. **(D)** Smoothed and fitted activation and inactivation curves of the low-threshold conductance used in the reduced 4-compartment model of a cortical layer V cell. The activation and inactivation curves obtained from experimental data was reduced to 30 data points by fitting to the nearest 5 mV value and then interpolated to 3000 points. The original curves have been left shifted by –6 mV in the model.

Figure 4 shows the activation, inactivation, and deinactivation variables based on the *in vitro* data. The activation curve (Figure 4A) was obtained by normalizing the low-threshold spike (LTS) amplitude for each depolarizing current stimulus to the maximal LTS amplitude of that cell. The initial activation of LTS occurred at −76 mV and complete activation at −42 mV with the greatest variability between neurons occurring in the linear range between −55 and −45 mV. The inactivation curve (Figure 4B) was obtained by normalizing the rebound potential at the end of each hyperpolarizing current prepulse to the maximum rebound potential for a neuron generated by the most hyperpolarized prepulse. The time course for deinactivation curve (Figure 4C) was obtained by eliciting hyperpolarizing pulses of constant amplitude from a holding potential of −40 mV and varying the width of the hyperpolarizing prepulse. The amplitude of the rebound potential was normalized to the maximum amplitude for that cell and plotted as a function of hyperpolarization time. The activation and inactivation curves for the rebound conductance obtained from the experimental data were reduced to 30 data points by a fit to the nearest 5 mV value in the range −90 to −40 mV. These data were used to interpolate a table of 3000 voltage states across that range to obtain the smoothed and fitted activation and inactivation curves (Figure 4D) to be used in the cortical neuron model.

### Matching *in vitro* data to single neuron model

Details of the reduced infragranular pyramidal cell model have been described in the Methods Section (also see cortical component CX in Figure 1). The model consisted of the following currents: fast sodium (I_Na_), potassium delayed rectifier (I_K_DR_), cortical low-threshold/rebound (I_CX_REB_), hyperpolarization-activated cation (I_H_), potassium after-hyperpolarization (I_AHP_), calcium dependent potassium (I_K[Ca]_), and the potassium A-current (I_A_). The channel description for the I_CX_REB_ current was based on *in vitro* data directly obtained from the activation and inactivation curves (Figure 4D). The experimentally determined activation and inactivation curves (Figures 4A,B) were reduced to 30 data points by “eyeball” fit to nearest 5 mV value from −90 to +5 mV. In our experimental dataset, this ranged from −80 to −40 mV and all values outside this range were set to 0 or 1. The GENESIS function *tweaktau* was then used to extrapolate the data to 3000 data points within the range −80 to −40 mV to obtain a smooth curve. The function *scaletabchan* was used to left shift the curves by −6 mV to give a more accurate depiction of channel properties and obtain realistic LTS and PIR responses.

An extensive parameter search was performed to obtain physiologically realistic responses to current stimulus protocols to demonstrate post-inhibitory rebound in the model. We found it was necessary to left shift the curves in the model by 6 mV (i.e., in a more hyperpolarized direction) to generate rebound that matched experimentally observed results. Figure 4D shows the final smoothed and shifted activation and inactivation curves for the I_CX_REB_ conductance implemented in the model. The maximal conductance values used were (in S/m^2^): *g*_K_DR_ = 200, *g*_CX_REB_ = 7, *g*_H_ = 12.55, *g*_AHP_ = 2.1, *g*_K[Ca]_ = 0.28, and *g*_A_ = 12. To determine rebound parameters in isolation, the fast Na^+^ maximal conductance was set to zero (*g*_*Na*_ = 0) to simulate the effect of the intracellular Na^+^ blocker QX-314.

**Figure 5:**
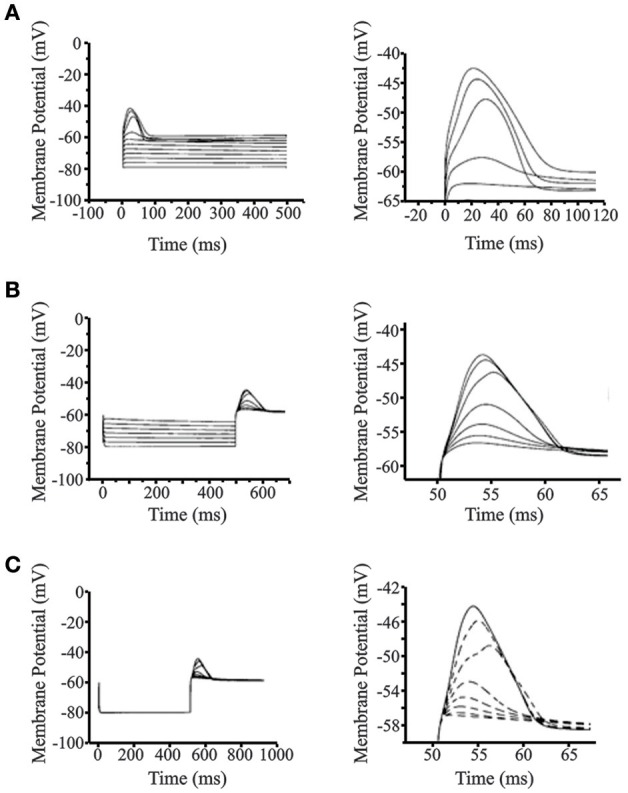
**Simulations of low-threshold spike (LTS) and post-inhibitory rebound (PIR) in cortical layer V cells**. **(A)** Simulated LTS response for increasing depolarizing 500 ms step pulses (left panel). Close up view of the LTS is seen on the right panel showing realistic responses compared to experimental data. The resting membrane potential was set at −80 mV and *g*_Na_ = 0 S/m^2^ to simulate action of QX-314 and block action potentials. **(B)** Simulated PIR response to increasing hyperpolarizing step pulses of 500 ms. Close up view of the PIR is seen on the right panel showing realistic PIR responses compared to experimental data. The resting membrane potential was set at −80 mV and *g*_Na_ = 0 S/m^2^ to simulate action of QX-314 and block action potentials. **(C)** Relationship between the low-threshold conductance channel (*g*_CX_REB_) implemented from experimental data and the post-inhibitory rebound response observed in the cortical cell model. The maximal value of *g*_CX_REB_ is varied from 0 to 7 S/m^2^. Close-up view of PIR response and its dependence to the low-threshold channel conductance (*g*_CX_REB_) is seen on the right panel. Figures on the right for **(A–C)** are drawn to different scale to highlight LTS and rebound response.

Figure 5 shows the simulated LTS and post-inhibitory rebound in the reduced 4-compartment cortical neuron model. With the membrane potential set at −80 mV and *g*Na = 0 (to simulate the action of QX-314), incremental LTS spikes and rebound potentials are observed with increasing depolarizing and hyperpolarizing current pulses, respectively (Figures 5A,B). The near all-or-nothing nature of the LTS evoked by stimuli between 0.7 and 0.8 nA reflects the steep slope of the activation curve. In these simulations, the maximum width of the LTS at its base was 70 ms which is close to the average value observed in the experimental data (67.5 ms). The maximum amplitude of the simulated LTS was 15 mV, near the average experimental value of 13.7 mV. The maximum rebound potential followed a hyperpolarizing current pulse current pulse of −0.7 nA which was well within the physiological range. The rebound width was 110 ms which exceeded the mean but was in the range of experimentally observed values. The amplitude of the rebound spike was close the mean observed value (Figure 5B). Figure 5C shows the increasing amplitude of rebound response to a constant hyperpolarizing current pulse of −0.8 nA with the maximal low-threshold conductance *g*_CX_REB_ varied from 0 to 7 S/m^2^. This finding shows that the post-inhibitory rebound response in the cortical cell model arises primarily as a result of the low-threshold conductance implemented from experimentally observed activation and inactivation data.

### Network model

**Figure 6:**
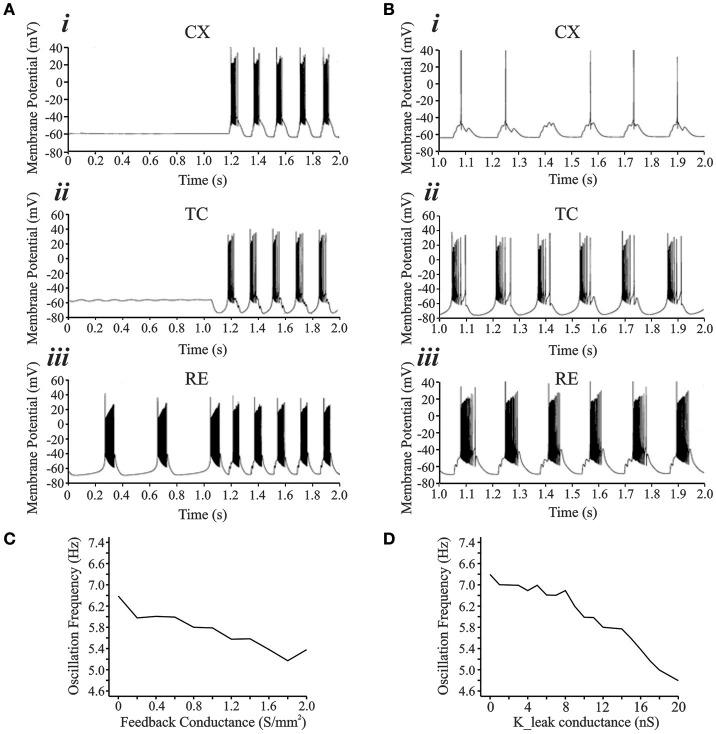
**(A)** Development of rhythmically bursting oscillations from an initial condition. **(A*i*,A*ii*,A*iii*)** show the activity from CX, TC, and RE cell, respectively. The RE cell **(A*iii*)** acts as the “initiator” cell oscillating at 2–3 Hz initially and subsequently entrains the entire network to oscillate at 6–7 Hz. **(B)** With *g*_CX_REB_ = 0, CX cell **(B*i*)** is not entrained into the bursting oscillatory network activity. Tonic single spikes occurred riding on top of summated EPSPs arriving from the TC cell onto the neck of the CX cell. TC **(B*ii*)** and RE **(B*iii*)** cells oscillate at 3 Hz frequency. **(C)** Increasing the strength of cortical feedback conductance to TC and RE causes a decrease in the frequency of oscillations. **(D)** Increase in the leak conductance value also causes decrease in oscillation frequency.

Details of the network model are outlined in the Methods Section. Figure 6A shows the development of oscillations in the interconnected network simulation (see Figure 1). For this simulation, the synaptic parameters were adjusted to keep the CX and TC cells quiescent while RE was bursting intrinsically at 2.5 Hz (Figures 6A*i–iii*). In the first second of simulation time, there are two bursts in the RE neuron whereas the TC and CX neurons show no spiking. This initial condition is based on the theory that a few RE or TC cells may be subgrouped as initiator cells that may start oscillating intrinsically and then gradually recruit other RE and TC cells as well as *in vivo* and computational data that show oscillations to be present in isolated RE nuclei (Steriade et al., 1987; Destexhe et al., 1994; Fuentealba et al., 2004). To simulate the RE initiator condition, the synaptic connectivity was removed between RE and TC cells for the initial 1 s period. Addition of inhibitory synaptic connectivity (RE → TC, g_GABA_ = 1 S/m^2^), produced hyperpolarization in the TC neuron leading to post-inhibitory rebound potential in the TC neuron along with burst spike activity. Burst spiking in the TC neuron also led to reciprocal excitatory EPSPs in the RE neuron and feed forward EPSPs in the CX neuron and the entire circuit oscillated at 6.5 Hz, which is in the lower range of spindle oscillations. Therefore, the RE initiator neurons entrained the entire TC-RE-CX circuit into the spindle oscillations frequency range. We also found that the low threshold conductance in the CX cell was essential for its rhythmic burst firing since setting g_CX_REB_ = 0 abolishes bursting activity in the CX neuron, though subthreshold oscillations remain (Figure 6B).

We also examined the effect of cortical feedback and resting thalamic leak conductances on the oscillation frequency. Across the range of excitatory corticothalamic synaptic conductances, the oscillation frequency reduced from 6.4 to 5.2 Hz for feedback ranging from 0 to 2 S/m^2^ (Figure 6C). For the simulations in Figures 6A,B, *g*_FEEDBACK_ was set at 0.4 S/m^2^. Figure 6D shows the effect of varying the TC resting potassium conductance value, *g*_LEAK_, on oscillation frequency. At the initial value of 11 nS, the frequency of oscillation was at the lower end of the spindle range of 6 Hz. Increasing the *g*_LEAK_ caused a decrease in the frequency, whereas decreasing *g*_LEAK_ caused an increase in the frequency. The range of frequencies was from 6.8 Hz at a *g*_LEAK_ value of 0 nS −4.8 Hz at a *g*_LEAK_ value 20 nS. Therefore, there is an inverse relationship between this passive potassium conductance value and the frequency of oscillation. Altering the *g*_LEAK_ conductance represents changes in the resting membrane potential of TC neurons that may arise from excitatory brainstem inputs during different physiological states (Bazhenov et al., 2002; Willis et al., 2015).

### Dynamical analysis

#### Phase plots

In a computational model, phase plots or phase portraits provide a way to plot multiple dynamic variables that vary with time (e.g., activation and inactivation gates of conductances, Ca^2+^ concentration, etc.) as a function of each other at the same time during continuously evolving neuronal behavior. These plots provide us with information about the dynamics of these variables that ultimately determine the evolution of the membrane potential.

**Figure 7:**
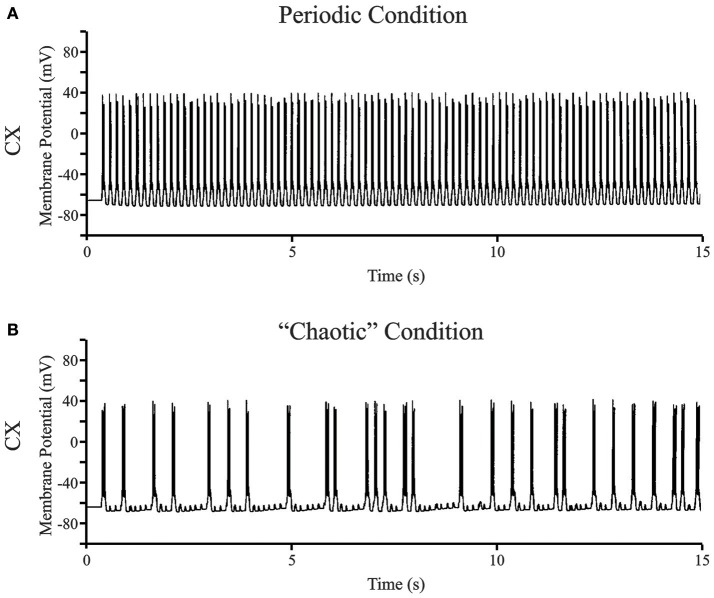
**(A)** Representative data set for the full bursting oscillations at 6–7 Hz in CX neuron. The periodic oscillations at 6–7 Hz were obtained by setting TC → CX synaptic excitation to 2.0 S/m^2^ and RE → TC, and synaptic inhibition to 1.0 S/m^2^. **(B)** The occurrence of bursts in the CX neuron visually appears irregular aperiodic in the transition region. The synaptic connections were set at TC → CX synaptic excitation: 1.4 S/m^2^ and RE → TC synaptic inhibition: 0.08 S/m^2^.

**Figure 8:**
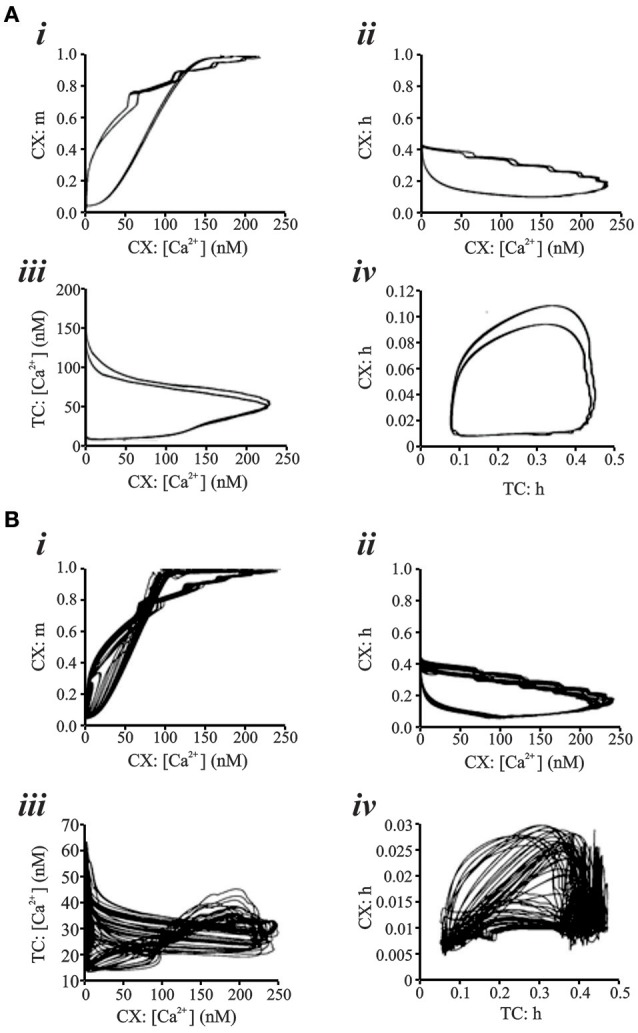
**(A)** Phase Plots for various parameters representing low-threshold conductance dynamics and Ca^2+^ concentration in CX and TC cell of network simulation. All phase plots exhibit a 2 period limit cycle. “Control” parameter settings are TC → CX synaptic excitation: 2.0 S/m^2^ and RE → TC synaptic inhibition: 1.0 S/m^2^. Computed Lyapunov Exponent is −0.05. **(B)** With TC → CX synaptic excitation: 1.6 S/m^2^ and RE → TC synaptic inhibition: 0.08 S/m^2^, phase plot analysis shows strange attractor dynamics for the same parameters as in **(A)**. Lyapunov Exponent is +0.24 and fractal dimension estimate from the Allan Factor time curve is 2.3.

Figure 7 shows two representative data sets for 15 s of simulated data in the putative chaotic condition and in the periodic condition for the CX neuron. The periodic oscillations at 6–7 Hz were obtained by setting TC → CX synaptic excitation to 2.0 S/m^2^ and RE → TC, and synaptic inhibition to 1.0 S/m^2^ (Figure 7A). The irregular aperiodic bursts observed in the putative transition region were obtained by setting TC → CX synaptic excitation: 1.4 S/m^2^ and RE → TC synaptic inhibition: 0.08 S/m^2^ (Figure 7B). To discern the underlying mechanisms between the two states, we constructed four phase plots for each of the periodic and putative chaotic conditions focusing on the Ca^2+^ concentration and the calcium channel activation/inactivation dynamics. These include (i) Internal Ca^2+^ concentration [Ca^2+^] of CX *vs*. low-threshold conductance (g_CX_REB_) activation variable (*m*) of CX, (ii) Internal Ca^2+^ concentration [Ca^2+^] of CX vs. low-threshold conductance (g_CX_REB_) inactivation variable (*h*) of CX, (iii) Internal Ca^2+^ concentration [Ca^2+^] of TC vs. Internal Ca^2+^ concentration [Ca^2+^] of CX, and (iv) low-threshold conductance (g_*T*_) inactivation variable (*h*) of TC vs. low-threshold conductance (g_CX_REB_) inactivation variable (*h*) of CX. All of the phase plots in Figures 8A*i–iv* are similar to each other because they all exhibit a strict period two limit cycle and represent a rhythmically oscillating state, similar to what is observed in sleep. The same variables are plotted against each other in Figures 8B*i–iv* in the putative chaotic state corresponding to the spike burst pattern observed in Figure 7B. In contrast to Figure 8A, these plots show a rich banded structure and overlapping trajectories which characterize the presence of strange attractors within a chaotic regime.

#### Bifurcation plot

**Figure 9:**
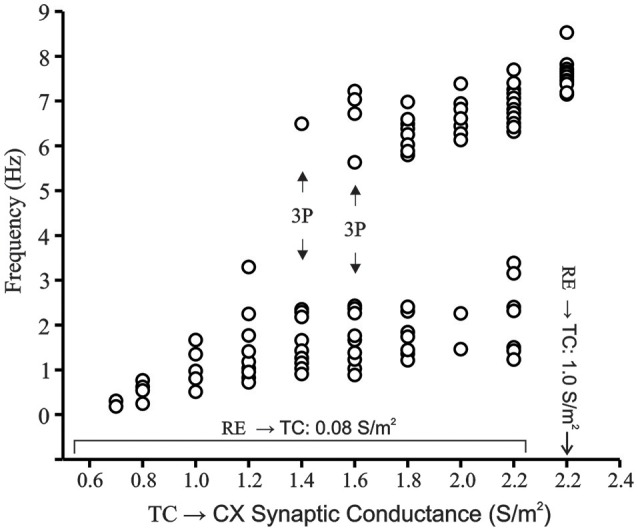
**Bifurcation plot of CX cell in network model in transition region**. RE → TC synaptic inhibition is set at 0.08 S/m^2^. Frequency of burst occurrences are plotted vs. increasing TC → CX synaptic excitation. Bursts are defined as groups of spikes with interspike intervals of less than 10 ms. On the x-axis, the point 2.2 represents a TC → CX synaptic excitation of 2.2 S/m^2^ and RE → TC synaptic inhibition of 0.08 S/m^2^ as well as TC → CX: 2.2 S/m^2^ and RE → TC: 1.0 S/m^2^ as distinct points on the x-scale. The latter represents the fully periodic oscillation state. Generation of apparent period-three cycle is indicated in the route to chaos.

Whereas, phase plots reveal information about the underlying dynamics by plotting variables against each other, bifurcation plots show the possible transition from periodic to aperiodic or chaotic states. Figure 9 shows the bifurcation plot of the instantaneous frequency of spikes (1/ISI) in Hz vs. the TC → CX excitatory synaptic conductance with RE → TC synaptic inhibition set at 0.08 S/m^2^. At low TC → CX values a single period is observed. The spread of points in the lower range increases (0.7–1.2 S/m^2^) until at 1.4 and 1.6 S/m^2^ where an apparent period-three limit occurs (indicated by arrow). This implies the presence of a chaotic regime (Li and Yorke, 1975; Canavier et al., 1990). Data points between TC → CX values of 1.4 and 2.2 S/m^2^ occupy a spectrum of values in both frequency ranges with no points in the middle region between 4 and 5.5 Hz. We have also shown the abscissa point TC → CX = 2.2 S/m^2^ with RE → TC synaptic inhibition increased to 1.0 S/m^2^ which is the periodic oscillatory bursting regime (see Figure 7A). At this point the lower range of frequencies abruptly disappears and only the higher frequency points remain with some spread in this range. Thus, the bifurcation plot demonstrates the route to chaos and back to periodicity.

#### Fano and Allan Factor

**Figure 10:**
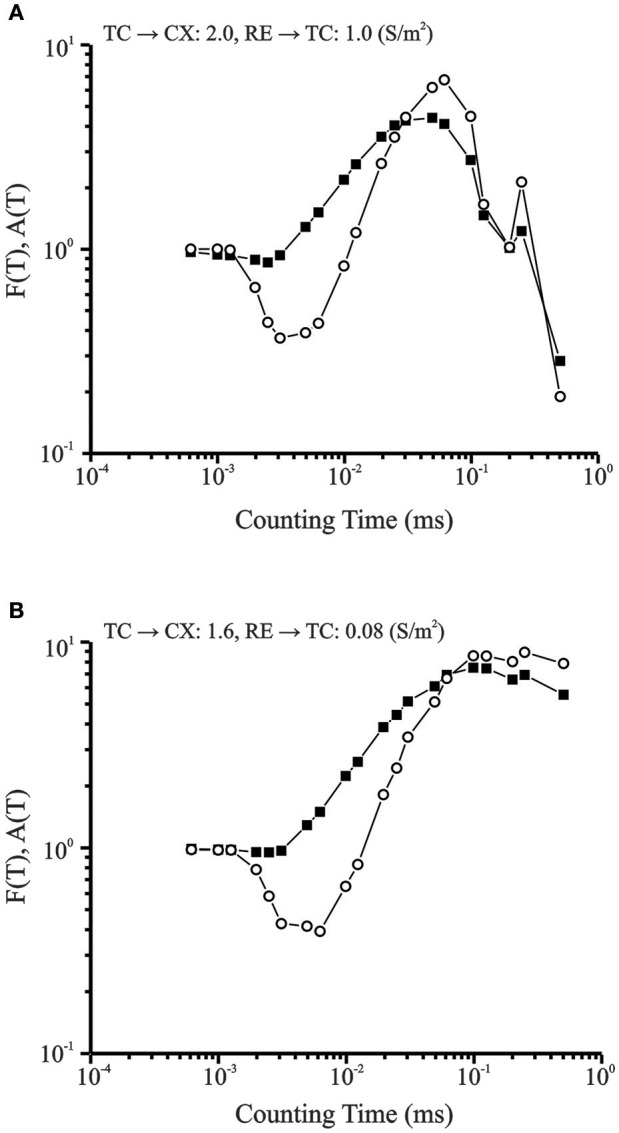
**(A)** Fano Factor (solid squares) and Allan Factor (open circles) time curve for data set in periodic condition. For longer counting times in the same range, the burst pattern has negative exponent values that indicate periodic behavior. Corresponding Lyapunov Exponent is −0.05. **(B)** Fano factor time curve for CX cell in transition region (solid data points). Power law growth for longer counting times indicate fractal nature of burst pattern. Allan Factor time curve is shown by line connecting open data points. The fractal dimension obtained from the slope of the Allan Factor curve was 2.3. Lyapunov Exponent is +0.24.

Fano factor and Allan Factor were computed for the chaotic and periodic conditions and plotted on a log-log scale (Figure 10). Both measures show power law growth between 10^0^ and 10^1^ for longer counting times which indicate fractal nature of burst occurrences (Lowen and Teich, 1996). The fractal dimensions estimated from the slope of these plots were 1.16 for the Fano Factor (solid squares) and 2.31 for the Allan Factor (open circles). For the periodic condition, at longer counting times in the same range, the burst pattern shows negative slope.

#### Lyapunov exponents

We calculated the Lyapunov exponents for the two data sets shown in Figure 7. With the variable measured being the membrane potential of the CX neuron, the data set representing the transition region the Lyapunov exponent value was computed to be +0.24 for 1000 iterations, which, being a value greater than zero, indicates a chaotic regime (Abarbanel et al., 1993). In contrast, in the periodic condition, a negative Lyapunov exponent value of −0.05 was obtained.

**Figure 11:**
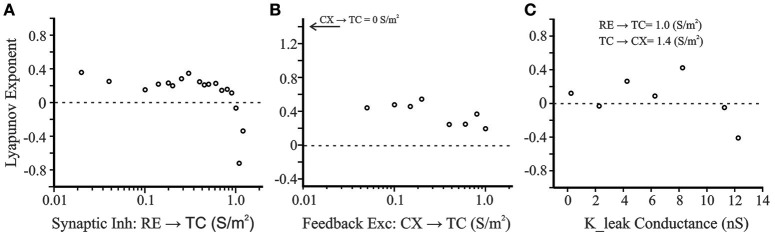
**(A)** Positive Lyapunov exponents are obtained for RE → TC inhibition 0.02 S/m^2^ to just below 1.0 S/m^2^. At 1.0 S/m^2^ and beyond the exponents abruptly become negative indicating a non-chaotic regime in this range. **(B)** Varying cortical feedback excitatory synaptic strength (CX → TC: 0.05–1 S/m^2^) produces LE values in the range 0.2–0.6. Removal of the cortical feedback loop (CX → TC = 0.0 S/m^2^) produces a larger LE of 1.4 (not shown in semilog scale). **(C)** Lyapunov Exponents as a function of a potassium leak conductance simulating excitatory brainstem neuromodulation of the thalamus. Decreasing *g*_LEAK_ simulates transition conditions from sleep to wakefulness and the system enters a chaotic regime.

Given the importance of RE → TC inhibition in generating post-inhibitory rebound and oscillatory states, we further characterized LE as a function of RE → TC synaptic inhibition (Figure 11A). With the CX membrane potential once again as the variable of interest, positive Lyapunov exponent values are obtained for RE → TC inhibition 0.02 S/m^2^ to just below 1.0 S/m^2^. At ≥1.0 S/m^2^ the Lyapunov exponents abruptly become negative indicating a transition to non-chaotic regime. Therefore, the cortical membrane potential demonstrates sensitivity to initial conditions between 0.02 and 1.0 S/m^2^, which is a hallmark of a chaotic system. Given the importance of the strength of CX → TC feedback on oscillation frequency (Figure 6C), we also examined Lyapunov exponents at varying CX → TC excitatory feedback synaptic strength while keeping other synaptic connectivity strengths constant (RE → TC = 0.08 S/m^2^; TC → CX = 1.4 S/m^2^). Varying CX → TC synaptic strength from 0.05 to 1 S/m^2^ produces Lyapunov exponents in the range 0.2–0.6 (Figure 11B). However, removal of the cortical feedback loop (CX → TC = 0.0 S/m^2^) produces a larger Lyapunov exponent of 1.4 (not shown in semilog scale in Figure 11B). In our previous simulations with only an interconnected TC and RE neurons, positive Lyapunov exponents were obtained in the range of 1–2 (Figure 5 in Paul et al., 1998). There is a wide spectrum of Lyapunov Exponent magnitudes reported in neural simulations with positive values in the range of 10^−4^ to show chaotic behavior in a single neuron model of R15 bursting cell of Aplysia (Canavier et al., 1990).

Figure 11C shows the Lyapunov exponents for the CX cell as a function of the potassium leak conductance (K_leak). Decreasing the leak conductance from its initial value simulates the effect of excitatory brainstem neuromodulatory input that accompanies the transition from sleep to wakefulness. The other control parameters are set at TC → CX = 1.4 S/m^2^ and RE → TC = 1.0 S/m^2^. With these settings, the LE value at initial *g*_LEAK_ of 11.25 nS is −0.05—only just negative. For lower *g*_LEAK_, Lyapunov exponents were positive (except at *g*_LEAK_ = 2.25 nS), suggesting that brainstem modulators, which modulate the state of arousal (Williams et al., 1994; Jones, 2003), may produce a transition from periodic to chaotic dynamics.

## Discussion

### Summary

A minimal network model consisting of one TC cell, one RE cell, and one CX cell was developed based in part on realistic biophysical parameters as well as measured values from physiological experiments. These cells were interconnected with realistic anatomical projections and implemented as previously hypothesized circuit for the neural basis of thalamocortical oscillations (Steriade et al., 1993a,b; Steriade, 2005; Contreras, 2014). The network model was constructed such that the RE cell acted as an initiator cell and subsequently entrained the TC and CX cells into a bursting rhythm. We observed that chaotic dynamics were present between thalamocortical states that accompany the physiological transition from wakefulness to drowsiness. The presence of chaos was dependent on a number of physiological parameters, such as the resting leak conductance of TC neurons, and the strength of thalamocortical transmission. These data add to the growing body of literature suggesting that chaotic dynamics are present in real and simulated neural systems (Canavier et al., 1990; Wang, 1994; van Vreeswijk and Sompolinsky, 1996; Siegel and Read, 2001; Bertschinger and Natschläger, 2004; Battaglia et al., 2007; Sussillo and Abbott, 2009; Rajan et al., 2010; Jia et al., 2012) and suggest that the transition between sleep and wakefulness may be characterized by chaotic dynamics. The limitations and implications of this study are discussed below.

### Nonlinear dynamical analysis

The present study examined the region between the full scale oscillation observed during sleep and drowsiness and the transition region characterized by irregular burst occurrences. Simulation runs with phase plots of various parameters representing the low-threshold conductance dynamics revealed the presence of strange attractors in the transition region, in contrast to a two-period limit cycle observed in the periodic condition. Previous studies have shown the presence of strange attractors in the transition region between beating and bursting modes in simple models that incorporate a single isolated neuron with biologically realistic conductances and a variable externally applied current that determines the state of the neuron (Plant and Kim, 1976; Canavier et al., 1990; Wang, 1994; van Vreeswijk and Sompolinsky, 1996; Carelli et al., 2005). Other studies have similarly shown the presence of a chaotic region in a pair of coupled oscillators (Paul et al., 1998; Iqbal et al., 2014). Paul et al. (1998) demonstrated a novel method of calculating Lyapunov Exponents which was possible within the GENESIS environment. We have now extended those findings to a more complex and anatomically realistic circuit with the addition of a cortical component and used multiple lines of analysis that converge on the finding that a chaotic regime occurs during the transition between wakefulness and sleep. In this study, we have taken a proposed hypothesized circuit for thalamocortical oscillations, experimentally determined the activation, and inactivation parameters of the critical post-inhibitory rebound conductance in the cortical component of the network model and then demonstrated the presence of a chaotic regime in the cortical neuron whose network dynamics are solely under the control of network synaptic input to CX neuron or output from CX neuron. The major synaptic strength being varied is the RE → TC inhibition. Therefore, with no direct externally applied current, we show that the CX neuron dynamics demonstrate the presence of strange attractors and a chaotic regime. Therefore, the thalamo-reticulo-cortical loop with its closed organization and architecture and multiple time scales, isolated from sensory inputs, may also generate chaotic complexity (Cauller, 2003). The physiological significance of this is elaborated further in the discussion of nonlinear dynamics in sensory processing.

In the bifurcation plot we show a route to chaos and back to period-one regime with an apparent period-three stable regime at TC → CX excitation strength of 1.4 and 1.6 S/m^2.^ The wide spread of points show multiple limit cycles within the putative chaotic regime and is clearly different from what is observed at very low TC → CX excitatory synaptic strength as well as when RE → TC inhibition is increased to 1.0 S/m^2^. Although the period-three orbit is somewhat arbitrary in our simulations, the wide spread of points within the transition region tend to fall into two distinct clumps and may be described as a bifurcation between dynamical states (Ciszak and Bellesi, 2011).

Presence of a period-three regime is known to be a hallmark of the transition to chaos (Canavier et al., 1990). Both the Fano Factor and the Allan Factor demonstrate a power law growth for longer counting times in the range 0–1 and indicate that the irregular burst pattern observed in the transition region is fractal in nature.

Finally, simulation runs were carried out to estimate the Lyapunov Exponent of the dynamical system. The Lyapunov Exponent measures the sensitivity of the dynamical system to just slightly different initial conditions. Positive Lyapunov exponents is considered to be the quintessential indicator of a chaotic regime (Abarbanel et al., 1993). Simulation runs in the representative transition region produced a positive Lyapunov exponent. In the periodic case, the Lyapunov Exponent was negative. All of these findings strongly indicate a chaotic regime in the transition region of network activity. These findings are consistent with a body of literature suggesting that sleep-wake transitions may be characterized by power law dynamics and as a bifurcation between different states (Lo et al., 2004; Chu-Shore et al., 2010; Ciszak and Bellesi, 2011), though this has not yet been measured using tools that provide resolution at the level of synaptic currents. In addition, it is well known that paroxysmal states such as seizures are more prone to occur during the sleep-wake transition (Da Silva et al., 1984; Coenen et al., 1991). These data suggest that the presence of strange attractors at the transition state allows rapid global transitions from one state to another (e.g., from wakefulness to sleep), but may also lead to rapid transitions to pathological states (e.g., seizures). Lo et al. (2004) noted power-law “fractal-like” behavior in sleep-wake transitions in mice and rats and have suggested that the transitions between sleep and wakefulness were not random fluctuations but related to underlying neural mechanisms of sleep control. The development of neural models for wakefulness-sleep transitions would be helpful for the understanding and regulation of sleep and wakefulness.

### Limitations of current study

The network model employed in the present study was minimal, in the sense that it consisted of one TC, one RE, and one CX cell, each with simplified morphologies, an approach based on the assumption that the behavior of large networks of neurons can be explained by the behavior of smaller networks of neurons. Clearly other elements could potentially influence the behavior of the model, such as dendritic calcium currents (Crandall et al., 2010), modifications of the calcium dynamics in RE and TC cells to reflect their physiological differences (Talley et al., 1999), or the addition of more cells to introduce potential cross-channel effects, due to either dendro-dendritic synapses, gap junctions, or nonreciprocal interactions between TC and RE cells (Deschênes et al., 1985; Pinault and Deschênes, 1998; Crabtree and Isaac, 2002; Landisman et al., 2002). Furthermore, in our model we do not distinguish between the diffuse matrix thalamocortical pathway projecting to the superficial layers of CX and the spatially selective core pathway which project to the granular layer (Piantoni et al., 2016). It is likely that such modifications would alter the quantitative behavior of the model, though it is not yet known if they would alter it qualitatively (i.e., would alter whether chaotic behavior would be observed). Despite these simplifications, we note that simple models have been used successfully to gain insights into network behavior (Grillner et al., 1988; Destexhe et al., 1993; Ching et al., 2010) though the ability of the current model to scale up to populations of neurons is yet unknown. Thus, a future version of this model could include multiple instances of each type of cell which may increase the validity of the model and provide opportunities to examine the impact(s) of the modifications described above.

### Experimental predictions made by the model

The results from the current study predict that spike trains in cortical and thalamic neurons should reflect chaotic dynamics during the transitions from the sleeping to the waking state, and that non-chaotic dynamics should be seen during sleep and during waking state. To determine if chaotic dynamics definitively exist in real spike trains would involve computation of Lyapunov exponents from time series data from an *in vivo* preparation, which is extremely difficult (but not impossible) to compute on real biological neurons. Therefore, the more feasible approach would be to determine if fractal patterns are seen in spike trains during the sleep-wake transition. However, difficult, it may still be possible to determine Lyapunov exponents from time series data based on reconstructed attractor maps and determining the long term evolution of vectors defined by points on separated by at atleast one mean orbital period (Wolf et al., 1985).

Another prediction made by the model is that corticothalamic feedback has significant effects on thalamocortical function, which could be measured. For example, in the current study, the effect of increasing g_FEEDBACK_ was a lowering of the network oscillation frequency (Figure 6C). In addition, weakening cortical feedback (to zero), elevated the Lyapunov exponent to 1.4, suggesting a greater tendency for chaotic dynamics to be seen. Strengthening or weakening corticothalamic feedback could be done using a range of modern genetic tools (Stroh et al., 2013; Denman and Contreras, 2015), and the effects of this manipulation can be measured on the firing properties of thalamic neurons during sleep-wake transitions.

Similarly, in the current study, there is an inverse relationship between the leak conductance value (g_LEAK_) and the frequency of oscillation (Figure 6D). Varying g_LEAK_ on the TC and RE cells simulates the effect of neuromodulatory input (e.g., cholinergics) on the thalamus during transition from sleep to wakefulness. Excitatory brainstem neuromodulatory input causes a block of K_LEAK_ channels thus, causing the membrane potential to become more depolarized (McCormick and Prince, 1987). This effect is simulated by reduction of g_LEAK_ which results in an increase of oscillation frequency. Such an effect should be readily testable using application of cholinergic or other modulators while measuring thalamocortical oscillation frequency.

### Nonlinear dynamics in sensory processing

In this study we have explored the chaotic nature of irregular aperiodic bursting that occurs in the transition from sleep to wakefulness. Irregular bursts may also occur during and due to sensory processing of stimuli in the thalamus and cortex (Krahe and Gabbiani, 2004; Desbordes et al., 2008), which must merge with the ongoing thalamocortical activity characterized by spontaneous tonic activity (Cauller, 2003). We hypothesize that the coupling of bottom-up sensory input bursts with top-down feedback may also constitute chaotic regimes with infinite limit cycles which allow an organism to quickly switch attention or respond to external inputs. This hypothesis may be tested in more detailed large scale neuronal models that incorporate a layered cortical structure.

Chaotic behavior has been previously obtained in minimal reciprocal models of neuron pairs with small changes in connection strength (Jackson et al., 1996; Paul et al., 1998). In the current study, we have shown that the simulation of a minimal thalamo-reticulo-cortical loop, isolated from sensory inputs, also generates chaotic complexity. Therefore, the thalamo-reticulo-cortical loop with its closed organization and architecture and multiple time scales, may also generate chaotic complexity with a system of multiple self-organized attractors and attractor sequences where each attractor space or unit refers to a particular spatio-temporal firing pattern (Cauller, 2003). From a physiological perspective, the cortex may contain specific instances self-organized attractor spaces with the rich complexity of chaotic dynamics which contain an internal model based on the sum of experiences of an organism. Top-down influences are continuously probed and tested by bottom up sensory inputs which may modify the attractor sequence. Even in the development phase a newborn infant spends much of its time in REM sleep which may correspond to dynamic self-organization of attractor spaces with chaotic complexity and is primed for further refinement as the infant begins to explore the environment (Cauller, 2003).

### Conclusions

The current data extend previous work showing that chaotic dynamics are present in neural spike trains at the transition points between stable states. Chaotic dynamics may permit rapid transitions between states—increasing behavioral and cognitive flexibility, possibly at the cost of transitions to pathological states, such as seizures. The use of a computational model permits quantification of certain values, such as the Lyapunov exponent, which are very difficult to compute in biological data. Calculation of the Lyapunov exponent permitted a systematic exploration of the impact of corticothalamic feedback and thalamic depolarization on chaotic behavior. This exploration will allow new hypotheses to be tested about the transition between sleeping and waking states.

## Author contributions

KP and LC designed the study. KP conducted electrophysiological experiments and developed computational models. KP, LC, and DL analyzed data and interpreted results. KP and DL prepared manuscript.

## Funding

This work was supported by the NIH grant NIDCD-R21DC014765 awarded to DL.

### Conflict of interest statement

The authors declare that the research was conducted in the absence of any commercial or financial relationships that could be construed as a potential conflict of interest.

## References

[B1] Abarbanel H. D. Brown R. Sidorowich J. J. Tsimring L. S. (1993). The analysis of observed chaotic data in physical systems. Rev. Mod. Phys. 65:1331 10.1103/RevModPhys.65.1331

[B2] Allan D. W. (1966). Statistics of atomic frequency standards. Proc. IEEE 54, 221–230. 10.1109/PROC.1966.4634

[B3] Amzica F. Steriade M. (1998). Electrophysiological correlates of sleep delta waves1. Electroencephalogr. Clin. Neurophysiol. 107, 69–83. 10.1016/S0013-4694(98)00051-0 9751278

[B4] Astori S. Wimmer R. D. Prosser H. M. Corti C. Corsi M. Liaudet N.. (2011). The CaV3. 3 calcium channel is the major sleep spindle pacemaker in thalamus. Proc. Natl. Acad. Sci. U.S.A. 108, 13823–13828. 10.1073/pnas.1105115108 21808016 PMC3158184

[B5] Barnes J. Allan D. (1966). A statistical model of flicker noise. Proc. IEEE 54, 176–178. 10.1109/PROC.1966.4630

[B6] Battaglia D. Brunel N. Hansel D. (2007). Temporal decorrelation of collective oscillations in neural networks with local inhibition and long-range excitation. Phys. Rev. Lett. 99:238106. 10.1103/PhysRevLett.99.238106 18233419

[B7] Bazhenov M. Timofeev I. Steriade M. Sejnowski T. J. (2002). Model of thalamocortical slow-wave sleep oscillations and transitions to activated states. J. Neurosci. 22, 8691–8704. 12351744 10.1523/JNEUROSCI.22-19-08691.2002 PMC6757797

[B8] Beierlein M. Connors B. W. (2002). Short-term dynamics of thalamocortical and intracortical synapses onto layer 6 neurons in neocortex. J. Neurophysiol. 88, 1924–1932. 12364518 10.1152/jn.2002.88.4.1924

[B9] Bertschinger N. Natschläger T. (2004). Real-time computation at the edge of chaos in recurrent neural networks. Neural Comput. 16, 1413–1436. 10.1162/089976604323057443 15165396

[B10] Bezdudnaya T. Cano M. Bereshpolova Y. Stoelzel C. R. Alonso J.-M. Swadlow H. A. (2006). Thalamic burst mode and inattention in the awake LGNd. Neuron 49, 421–432. 10.1016/j.neuron.2006.01.010 16446145

[B11] Bower J. M. Beeman D. (1998). The Book of GENESIS: Exploring Realistic Neural Models with the General Neural Simulation System. Santa Clara, CA: TELOS.

[B12] Canavier C. Clark J. Byrne J. (1990). Routes to chaos in a model of a bursting neuron. Biophys. J. 57, 1245. 10.1016/s0006-3495(90)82643-6 1697484 PMC1280834

[B13] Canavier C. C. Shepard P. D. (2009). Chaotic versus stochastic dynamics: a critical look at the evidence for nonlinear sequence dependent structure in dopamine neurons. J. Neural. Transm. Suppl. 121–128. 20411772 10.1007/978-3-211-92660-4_9 PMC4434592

[B14] Carelli P. V. Reyes M. B. Sartorelli J. C. Pinto R. D. (2005). Whole cell stochastic model reproduces the irregularities found in the membrane potential of bursting neurons. J. Neurophysiol. 94, 1169–1179. 10.1152/jn.00070.2005 15800078

[B15] Cauller L. (2003). The neurointeractive paradigm: dynamical mechanics and the emergence of higher cortical function, in Computational Models for neuroscience, eds Hecht-Nielsen R. Mckenna T. (London: Springer-Verlag), 1–23.

[B16] Ching S. Cimenser A. Purdon P. L. Brown E. N. Kopell N. J. (2010). Thalamocortical model for a propofol-induced alpha-rhythm associated with loss of consciousness. Proc. Natl. Acad. Sci. U.S.A. 107, 22665–22670. 10.1073/pnas.1017069108 21149695 PMC3012501

[B17] Chu-Shore J. Westover M. B. Bianchi M. T. (2010). Power law versus exponential state transition dynamics: application to sleep-wake architecture. PLoS ONE 5:e14204. 10.1371/journal.pone.0014204 21151998 PMC2996311

[B18] Ciszak M. Bellesi M. (2011). Synaptic plasticity modulates autonomous transitions between waking and sleep states: insights from a Morris-Lecar model. Chaos 21, 043119. 10.1063/1.3657381 22225356

[B19] Coenen A. M. Drinkenburg W. H. Peeters B. W. Vossen J. M. Van Luijtelaar E. L. (1991). Absence epilepsy and the level of vigilance in rats of the WAG/Rij strain. Neurosci. Biobehav. Rev. 15, 259–263. 10.1016/S0149-7634(05)80005-3 1906586

[B20] Contreras D. (2014). Low-frequency oscillations (anesthesia and sleep): overview, in Encyclopedia of Computational Neuroscience, eds Jaeger D. Jung R. (New York, NY: Springer-Verlag), 1–7.

[B21] Contreras D. Steriade M. (1995). Cellular basis of EEG slow rhythms: a study of dynamic corticothalamic relationships. J. Neurosci. 15, 604–622. 7823167 10.1523/JNEUROSCI.15-01-00604.1995 PMC6578315

[B22] Crabtree J. W. Isaac J. T. (2002). New intrathalamic pathways allowing modality-related and cross-modality switching in the dorsal thalamus. J. Neurosci. 22, 8754–8761. 12351751 10.1523/JNEUROSCI.22-19-08754.2002 PMC6757787

[B23] Crandall S. R. Govindaiah G. Cox C. L. (2010). Low-threshold Ca2+ current amplifies distal dendritic signaling in thalamic reticular neurons. J. Neurosci. 30, 15419–15429. 10.1523/JNEUROSCI.3636-10.2010 21084598 PMC3075467

[B24] Darbin O. Soares J. Wichmann T. (2006). Nonlinear analysis of discharge patterns in monkey basal ganglia. Brain Res. 1118, 84–93. 10.1016/j.brainres.2006.08.027 16989784

[B25] Da Silva A. M. Aarts J. Binnie C. Laxminarayan R. Da Silva F. L. Meijer J.. (1984). The circadian distribution of interictal epileptiform EEG activity. Electroencephalogr. Clin. Neurophysiol. 58, 1–13. 10.1016/0013-4694(84)90195-0 6203698

[B26] Denman D. J. Contreras D. (2015). Complex effects on *in vivo* visual responses by specific projections from mouse cortical layer 6 to dorsal lateral geniculate nucleus. J. Neurosci. 35, 9265–9280. 10.1523/JNEUROSCI.0027-15.2015 26109652 PMC4478248

[B27] Desbordes G. Jin J. Weng C. Lesica N. A. Stanley G. B. Alonso J. M. (2008). Timing precision in population coding of natural scenes in the early visual system. PLoS Biol. 6:e324. 10.1371/journal.pbio.0060324 19090624 PMC2602720

[B28] Deschênes M. Madariaga-Domich A. Steriade M. (1985). Dendrodendritic synapses in the cat reticularis thalami nucleus: a structural basis for thalamic spindle synchronization. Brain Res. 334, 165–168. 10.1016/0006-8993(85)90580-3 2986779

[B29] Destexhe A. Contreras D. Sejnowski T. J. Steriade M. (1994). A model of spindle rhythmicity in the isolated thalamic reticular nucleus. J. Neurophysiol. 72, 803–818. 7527077 10.1152/jn.1994.72.2.803

[B30] Destexhe A. McCormick D. A. Sejnowski T. J. (1993). A model for 8-10 Hz spindling in interconnected thalamic relay and reticularis neurons. Biophys. J. 65, 2473–2477. 10.1016/S0006-3495(93)81297-9 8312485 PMC1225988

[B31] Fuentealba P. Timofeev I. Steriade M. (2004). Prolonged hyperpolarizing potentials precede spindle oscillations in the thalamic reticular nucleus. Proc. Natl. Acad. Sci. U.S.A. 101, 9816–9821. 10.1073/pnas.0402761101 15210981 PMC470757

[B32] Gebber G. L. Orer H. S. Barman S. M. (2006). Fractal noises and motions in time series of presympathetic and sympathetic neural activities. J. Neurophysiol. 95, 1176–1184. 10.1152/jn.01021.2005 16306172

[B33] Grillner S. Buchanan J. T. Lansner A. (1988). Simulation of the segmental burst generating network for locomotion in lamprey. Neurosci. Lett. 89, 31–35. 10.1016/0304-3940(88)90476-4 3399139

[B34] Halassa M. M. Siegle J. H. Ritt J. T. Ting J. T. Feng G. Moore C. I. (2011). Selective optical drive of thalamic reticular nucleus generates thalamic bursts and cortical spindles. Nat. Neurosci. 14, 1118–1120. 10.1038/nn.2880 21785436 PMC4169194

[B35] Hill S. Tononi G. (2005). Modeling sleep and wakefulness in the thalamocortical system. J. Neurophysiol. 93, 1671–1698. 10.1152/jn.00915.2004 15537811

[B36] Huguenard J. R. McCormick D. A. (2007). Thalamic synchrony and dynamic regulation of global forebrain oscillations. Trends Neurosci. 30, 350–356. 10.1016/j.tins.2007.05.007 17544519

[B37] Iqbal M. Rehan M. Khaliq A. Hong K.-S. (2014). Synchronization of coupled different chaotic FitzHugh-Nagumo neurons with unknown parameters under communication-direction-dependent coupling. Comput. Math. Methods Med. 2014, 1–12. 10.1155/2014/367173 25101140 PMC4101220

[B38] Jackson M. E. Patterson J. Cauller L. J. (1996). Dynamical analysis of spike trains in a simulation of reciprocally connected ‘chaoscillators’: dependence of spike pattern fractal dimension on strength of feedback connections, in Computational Neuroscience: Trends in Research, ed Bower J. M. (Academic Press), 209–214.

[B39] Jahnsen H. Llinas R. (1984). Ionic basis for the electro-responsiveness and oscillatory properties of guinea-pig thalamic neurones *in vitro*. J. Physiol. (Lond). 349, 227–247. 10.1113/jphysiol.1984.sp015154 6737293 PMC1199335

[B40] Jia B. Gu H. Li L. Zhao X. (2012). Dynamics of period-doubling bifurcation to chaos in the spontaneous neural firing patterns. Cogn. Neurodyn. 6, 89–106. 10.1007/s11571-011-9184-7 23372622 PMC3253168

[B41] Jones B. E. (2003). Arousal Systems. Front. Biosci. 8, s438–s451. 10.2741/1074 12700104

[B42] Khazipov R. Sirota A. Leinekugel X. Holmes G. L. Ben-Ari Y. Buzsaki G. (2004). Early motor activity drives spindle bursts in the developing somatosensory cortex. Nature 432, 758–761. 10.1038/nature03132 15592414

[B43] Kim D. Song I. Keum S. Lee T. Jeong M.-J. Kim S.-S.. (2001). Lack of the burst firing of thalamocortical relay neurons and resistance to absence seizures in mice lacking α 1G T-type Ca 2+ channels. Neuron 31, 35–45. 10.1016/S0896-6273(01)00343-9 11498049

[B44] Koch C. (1999). Stochastic models of single cells, in Biophysics of Computation: Information Processing in Single Neurons, ed Stryker M. (New York, NY: Oxford University Press) 350–373.

[B45] Krahe R. Gabbiani F. (2004). Burst firing in sensory systems. Nat. Rev. Neurosci. 5, 13–23. 10.1038/nrn1296 14661065

[B46] Kramer M. A. Roopun A. K. Carracedo L. M. Traub R. D. Whittington M. A. Kopell N. J. (2008). Rhythm generation through period concatenation in rat somatosensory cortex. PLoS Comput. Biol. 4:e1000169. 10.1371/journal.pcbi.1000169 18773075 PMC2518953

[B47] Landisman C. E. Long M. A. Beierlein M. Deans M. R. Paul D. L. Connors B. W. (2002). Electrical synapses in the thalamic reticular nucleus. J. Neurosci. 22, 1002–1009. 11826128 10.1523/JNEUROSCI.22-03-01002.2002 PMC6758490

[B48] Li T.-Y. Yorke J. A. (1975). Period three implies chaos. Am. Math. Month. 82, 985–992. 10.2307/2318254

[B49] Liu X. B. Jones E. G. (1999). Predominance of corticothalamic synaptic inputs to thalamic reticular nucleus neurons in the rat. J. Comp. Neurol. 414, 67–79. 10494079

[B50] Llano D. A. Sherman S. M. (2008). Evidence for nonreciprocal organization of the mouse auditory thalamocortical−corticothalamic projection systems. J. Comp. Neurol. 507, 1209–1227. 10.1002/cne.21602 18181153

[B51] Lo C.-C. Chou T. Penzel T. Scammell T. E. Strecker R. E. Stanley H. E.. (2004). Common scale-invariant patterns of sleep–wake transitions across mammalian species. Proc. Natl. Acad. Sci. U.S.A. 101, 17545–17548. 10.1073/pnas.0408242101 15583127 PMC536051

[B52] Lowen S. B. Teich M. C. (1996). The periodogram and Allan variance reveal fractal exponents greater than unity in auditory−nerve spike trains. J. Acoust. Soc. Am. 99, 3585–3591. 10.1121/1.414979 8655790

[B53] Lüthi A. McCormick D. A. (1998). H-Current: properties of a neuronal and network pacemaker. Neuron 21, 9–12. 10.1016/S0896-6273(00)80509-7 9697847

[B54] McCormick D. A. Prince D. A. (1987). Actions of acetylcholine in the guinea−pig and cat medial and lateral geniculate nuclei, *in vitro*. J. Physiol. (Lond). 392, 147–165. 10.1113/jphysiol.1987.sp016774 2833597 PMC1192298

[B55] Meeren H. K. Pijn J. P. M. Van Luijtelaar E. L. Coenen A. M. da Silva F. H. L. (2002). Cortical focus drives widespread corticothalamic networks during spontaneous absence seizures in rats. J. Neurosci. 22, 1480–1495. 11850474 10.1523/JNEUROSCI.22-04-01480.2002 PMC6757554

[B56] Meyer H. S. Wimmer V. C. Hemberger M. Bruno R. M. de Kock C. P. J. Frick A.. (2010). Cell type–specific thalamic innervation in a column of rat vibrissal cortex. Cereb. Cortex. 20, 2287–2303. 10.1093/cercor/bhq069 20534783 PMC2936808

[B57] Pape H.-C. (1996). Queer current and pacemaker: the hyperpolarization-activated cation current in neurons. Annu. Rev. Physiol. 58, 299–327. 8815797 10.1146/annurev.ph.58.030196.001503

[B58] Paul K. Jackson M. Cauller L. J. (1998). Presence of a chaotic region between subthreshold oscillations and rhythmic bursting in a simulation of thalamocortical relay and reticular neurons, in Computational Neuroscience: Trends in Research, ed Bower J. M. (Academic Press), 95–100.

[B59] Piantoni G. Halgren E. Cash S. S. (2016). The contribution of thalamocortical core and matrix pathways to sleep spindles. Neural Plast. 2016:3024342. 10.1155/2016/3024342 27144033 PMC4842069

[B60] Pinault D. (2004). The thalamic reticular nucleus: structure, function and concept. Brain Res. Rev. 46, 1–31. 10.1016/j.brainresrev.2004.04.008 15297152

[B61] Pinault D. Deschênes M. (1998). Anatomical evidence for a mechanism of lateral inhibition in the rat thalamus. Eur. J. Neurosci. 10, 3462–3469. 10.1046/j.1460-9568.1998.00362.x 9824459

[B62] Plant R. Kim M. (1976). Mathematical description of a bursting pacemaker neuron by a modification of the Hodgkin-Huxley equations. Biophys. J. 16, 227. 10.1016/S0006-3495(76)85683-4 1252578 PMC1334834

[B63] Polack P.-O. Guillemain I. Hu E. Deransart C. Depaulis A. Charpier S. (2007). Deep layer somatosensory cortical neurons initiate spike-and-wave discharges in a genetic model of absence seizures. J. Neurosci. 27, 6590–6599. 10.1523/JNEUROSCI.0753-07.2007 17567820 PMC6672429

[B64] Rajan K. Abbott L. Sompolinsky H. (2010). Stimulus-dependent suppression of chaos in recurrent neural networks. Phys. Rev. E 82:011903. 10.1103/PhysRevE.82.011903 20866644 PMC10683875

[B65] Rall W. (1967). Distinguishing theoretical synaptic potentials computed for different soma-dendritic distributions of synaptic input. J. Neurophysiol. 30, 1138–1168. 6055351 10.1152/jn.1967.30.5.1138

[B66] Ramcharan E. J. Gnadt J. W. Sherman S. M. (2000). Burst and tonic firing in thalamic cells of unanesthetized, behaving monkeys. Vis. Neurosci. 17, 55–62. 10.1017/S0952523800171056 10750826

[B67] Rosanova M. Timofeev I. (2005). Neuronal mechanisms mediating the variability of somatosensory evoked potentials during sleep oscillations in cats. J. Physiol. (Lond). 562, 569–582. 10.1113/jphysiol.2004.071381 15528249 PMC1665518

[B68] Scharf R. Meesmann M. Boese J. Chialvo D. R. Kniffki K.-D. (1995). General relation between variance-time curve and power spectral density for point processes exhibiting 1/f β-fluctuations, with special reference to heart rate variability. Biol. Cybern. 73, 255–263. 10.1007/BF00201427 7548313

[B69] Sherman S. M. (2001). Tonic and burst firing: dual modes of thalamocortical relay. Trends Neurosci. 24, 122–126. 10.1016/S0166-2236(00)01714-8 11164943

[B70] Siegel R. M. Read H. L. (2001). Deterministic dynamics emerging from a cortical functional architecture. Neural Netw. 14, 697–713. 10.1016/S0893-6080(01)00045-4 11665764

[B71] Slater B. J. Willis A. M. Llano D. A. (2013). Evidence for layer-specific differences in auditory corticocollicular neurons. Neuroscience 229, 144–154. 10.1016/j.neuroscience.2012.10.053 23137545 PMC3534900

[B72] Stafstrom C. E. Schwindt P. C. Flatman J. Crill W. E. (1984). Properties of subthreshold response and action potential recorded in layer V neurons from cat sensorimotor cortex *in vitro*. J. Neurophysiol. 52, 244–263. 6090604 10.1152/jn.1984.52.2.244

[B73] Steriade M. (2005). Sleep, epilepsy and thalamic reticular inhibitory neurons. Trends Neurosci. 28, 317–324. 10.1016/j.tins.2005.03.007 15927688

[B74] Steriade M. Contreras D. Dossi R. C. Nunez A. (1993a). The slow (< 1 Hz) oscillation in reticular thalamic and thalamocortical neurons: scenario of sleep rhythm generation in interacting thalamic and neocortical networks. J. Neurosci. 13, 3284–3299. 8340808 10.1523/JNEUROSCI.13-08-03284.1993 PMC6576531

[B75] Steriade M. Domich L. Oakson G. Deschenes M. (1987). The deafferented reticular thalamic nucleus generates spindle rhythmicity. J. Neurophysiol. 57, 260–273. 3559675 10.1152/jn.1987.57.1.260

[B76] Steriade M. McCormick D. A. Sejnowski T. J. (1993b). Thalamocortical oscillations in the sleeping and aroused brain. Science 262, 679–685. 8235588 10.1126/science.8235588

[B77] Steriade M. Nunez A. Amzica F. (1993c). A novel slow (< 1 Hz) oscillation of neocortical neurons *in vivo*: depolarizing and hyperpolarizing components. J. Neurosci. 13, 3252–3265. 8340806 10.1523/JNEUROSCI.13-08-03252.1993 PMC6576541

[B78] Stroh A. Adelsberger H. Groh A. Rühlmann C. Fischer S. Schierloh A.. (2013). Making waves: initiation and propagation of corticothalamic Ca 2+ waves *in vivo*. Neuron 77, 1136–1150. 10.1016/j.neuron.2013.01.031 23522048

[B79] Sussillo D. Abbott L. F. (2009). Generating coherent patterns of activity from chaotic neural networks. Neuron 63, 544–557. 10.1016/j.neuron.2009.07.018 19709635 PMC2756108

[B80] Talley E. M. Cribbs L. L. Lee J.-H. Daud A. Perez-Reyes E. Bayliss D. A. (1999). Differential distribution of three members of a gene family encoding low voltage-activated (T-type) calcium channels. J. Neurosci. 19, 1895–1911. 10066243 10.1523/JNEUROSCI.19-06-01895.1999 PMC6782581

[B81] Teich M. C. (1989). Fractal character of the auditory neural spike train. IEEE Trans. Biomed. Eng. 36, 150–160. 10.1109/10.16460 2921061

[B82] Teich M. C. Heneghan C. Lowen S. B. Ozaki T. Kaplan E. (1997). Fractal character of the neural spike train in the visual system of the cat. JOSA A 14, 529–546. 10.1364/JOSAA.14.000529 9058948

[B83] Timofeev I. Bazhenov M. Sejnowski T. Steriade M. (2002). Cortical hyperpolarization-activated depolarizing current takes part in the generation of focal paroxysmal activities. Proc. Natl. Acad. Sci. U.S.A. 99, 9533–9537. 10.1073/pnas.132259899 12089324 PMC123175

[B84] van Vreeswijk C. Sompolinsky H. (1996). Chaos in neuronal networks with balanced excitatory and inhibitory activity. Science 274, 1724–1726. 10.1126/science.274.5293.1724 8939866

[B85] Wang X.-J. (1994). Multiple dynamical modes of thalamic relay neurons: rhythmic bursting and intermittent phase-locking. Neuroscience 59, 21–31. 10.1016/0306-4522(94)90095-7 8190268

[B86] Williams J. A. Comisarow J. Day J. Fibiger H. C. Reiner P. B. (1994). State-dependent release of acetylcholine in rat thalamus measured by *in vivo* microdialysis. J. Neurosci. 14, 5236–5242. 8083733 10.1523/JNEUROSCI.14-09-05236.1994 PMC6577063

[B87] Willis A. M. Slater B. J. Gribkova E. D. Llano D. A. (2015). Open-loop organization of thalamic reticular nucleus and dorsal thalamus: a computational model. J. Neurophysiol. 114, 2353–2367. 10.1152/jn.00926.2014 26289472 PMC4620136

[B88] Wolf A. Swift J. B. Swinney H. L. Vastano J. A. (1985). Determining Lyapunov exponents from a time series. Phys. D 16, 285–317. 10.1016/0167-2789(85)90011-9

[B89] Zikopoulos B. Barbas H. (2006). Prefrontal projections to the thalamic reticular nucleus form a unique circuit for attentional mechanisms. J. Neurosci. 26, 7348–7361. 10.1523/JNEUROSCI.5511-05.2006 16837581 PMC6674204

